# Supplementary material for: Investigating the Role of Maintenance TMS Protocols for Major Depression: Systematic Review and Future Perspectives for Personalized Interventions
Source: J Pers Med. 2023 Apr 21;13(4):697. doi: 10.3390/jpm13040697 (PMC10141590; doi:10.3390/jpm13040697)
Supplement: Supplementary file 1 [file jpm-13-00697-s001.zip › jpm-2332002-supplementary.pdf]

Table S1. Case report --- JBI **Case Reports Critical Appraisal Tool**[i]

Name	1	2	3	4	5	6	7	8
Langguth_2006	Y	Y	Y	Y	Y	Y	N	Y
Chatterjee,B_2012	Y	Y	Y	Y	Y	Y	Y	Y

Table S2. Case series --- JBI **Case Series Critical Appraisal Tool**[ii]

	1	2	3	4	5	6	7	8	9	10
O'Reardon_2005	Y	Y	Y	Y	Y	Y	Y	Y	Y	Y

Table S3. RCT- Risk of bias tools 2[iii]

	1	2	3	4	5	Overall Bias
Levkovitz,y_2015	**Low**	**Low**	**Low**	**Low**	**Low**	**Low**
Benadhira,R_2017	**Some concern**	**Low**	**Low**	**Low**	**Low**	**Some concern**
Wang, H.-N.2015	**Low**	**Some concern**	**Low**	**Low**	**Low**	**Some concern**

Table S4. Not RCT: **Robin-1**[iv]

	1	2	3	4	5	6	7	Overall Bias
Harel_2014	Serious	Low	Low	Low	Moderate	Serious	Low	Serious
Philip,N.S_2016	Low	Low	Low	Low	Moderate	Moderate	Low	Moderate
Fitzgerald_2013	Low	Low	Low	Low	Low	Low	Low	Low
Haesebaert. F_ 2014	Low	Low	Low	Low	Low	Low	Low	Low
Pridmore, S_2018 A	Moderate	Low	Low	Low	Low	Serious	Low	Serious
Pridmore, S_2018 B	Moderate	Low	Low	Low	Low	Serious	Low	Serious
Ricchieri_2013	Low	Low	Low	Low	Low	Moderate	Low	Moderate

---

[i] The Joanna Briggs Institute, Lockwood C, Porrit K, Munn Z, Rittenmeyer L, Salmond S, Bjerrum M, Loveday H, Carrier J, Stannard D. Aromataris E, Munn Z. Appendix 7.4 Critical appraisal checklist for case reports ***Joanna Briggs Institute Reviewer's Manual*** 2017.

[ii] The Joanna Briggs Institute, Lockwood C, Porrit K, Munn Z, Rittenmeyer L, Salmond S, Bjerrum M, Loveday H, Carrier J, Stannard D. Aromataris E, Munn Z. Appendix 7.3 Critical appraisal checklists for case series***Joanna Briggs Institute Reviewer's Manual*** 2017.

[iii] Sterne JAC, Savović J, Page MJ, Elbers RG, Blencowe NS, Boutron I, Cates CJ, Cheng H-Y, Corbett MS, Eldridge SM, Hernán MA, Hopewell S, Hróbjartsson A, Junqueira DR, Jüni P, Kirkham JJ, Lasserson T, Li T, McAleenan A, Reeves BC, Shepperd S, Shrier I, Stewart LA, Tilling K, White IR, Whiting PF, Higgins JPT. RoB 2: a revised tool for assessing risk of bias in randomised trials. *BMJ* 2019; **366**: l4898.

[iv] Sterne JAC, Hernán MA, Reeves BC, Savović J, Berkman ND, Viswanathan M, Henry D, Altman DG, Ansari MT, Boutron I, Carpenter JR, Chan AW, Churchill R, Deeks JJ, Hróbjartsson A, Kirkham J, Jüni P, Loke YK, Pigott TD, Ramsay CR, Regidor D, Rothstein HR, Sandhu L, Santaguida PL, Schünemann HJ, Shea B, Shrier I, Tugwell P, Turner L, Valentine JC, Waddington H, Waters E, Wells GA, Whiting PF, Higgins JPT. ROBINS-I: a tool for assessing risk of bias in non-randomized studies of interventions. BMJ 2016; 355; i4919; doi: 10.1136/bmj.i4919.
